# Cell-Cell Contact Preserves Cell Viability via Plakoglobin

**DOI:** 10.1371/journal.pone.0027064

**Published:** 2011-10-28

**Authors:** Qi Wei, Venkatesh Hariharan, Hayden Huang

**Affiliations:** Department of Biomedical Engineering, Columbia University, New York, New York, United States of America; Cedars-Sinai Medical Center, United States of America

## Abstract

Control over cell viability is a fundamental property underlying numerous physiological processes. Cell spreading on a substrate was previously demonstrated to be a major factor in determining the viability of individual cells. In multicellular organisms, cell-cell contact is likely to play a significant role in regulating cell vitality, but its function is easily masked by cell-substrate interactions, thus remains incompletely characterized. In this study, we show that suspended immortalized human keratinocyte sheets with persisting intercellular contacts exhibited significant contraction, junctional actin localization, and reinforcement of cell-cell adhesion strength. Further, cells within these sheets remain viable, in contrast to trypsinized cells suspended without either cell-cell or cell-substrate contact, which underwent apoptosis at high rates. Suppression of plakoglobin weakened cell-cell adhesion in cell sheets and suppressed apoptosis in suspended, trypsinized cells. These results demonstrate that cell-cell contact may be a fundamental control mechanism governing cell viability and that the junctional protein plakoglobin is a key regulator of this process. Given the near-ubiquity of plakoglobin in multicellular organisms, these findings could have significant implications for understanding cell adhesion, modeling disease progression, developing therapeutics and improving the viability of tissue engineering protocols.

## Introduction

Cell-cell interactions, which occur via specialized adhesion structures in cell junctions, regulate a variety of functions in multicellular organisms, including differentiation, barrier formation, tissue function and signal transduction [1], [2]. Despite these critical roles, cell-cell signaling is generally difficult to isolate from cell-substrate interactions, with the result that the latter has been studied more extensively. For example, many studies demonstrate that cell membrane receptors that mediate cell adhesion to the extracellular matrix (ECM) play a central role in sensing external mechanical stimuli, such as fluid shear stress, and transduce these signals into downstream intracellular changes [3], [4], [5], [6], [7]. One key finding is that cell viability is controlled via geometric factors, being dependent on cell spreading but not contact area per se [8]. Thus, cell-substrate adhesion is one critical regulator of cell life. Whether adhesion to other cells is important is the central question of this study.

Recent work has emphasized the multiple critical roles that cell-cell junctional proteins play in regulating the various facets of development, life and disease. For example, recent studies in arrhythmogenic right ventricular cardiomyopathy (ARVC) demonstrate that mutations in desmosomal proteins are thought to lead to alterations in cardiac and sometimes, dermal tissues. In particular, nuclear localization of the desmosomal protein plakoglobin is thought to lead to apoptosis [9], suggesting a role for junctional proteins in establishing or maintaining cell vitality.

The exact role of cell junctions and the relative impact of cell-substrate versus cell-cell interactions in preserving cell life remain unclear. In particular, it is clear that cells can, at least transiently, remain viable when plated sparsely (maintaining cell-substrate contact with spreading but having minimal or no cell-cell contact). However, normal culture conditions typically rely on establishing cell-cell contact and in fact, some cells are not viable when lacking cell-cell contact, even when appropriate substrate is plentiful [10]. We hypothesize that cell-cell contact is a fundamental regulator of cell viability, and based on ARVC-related observations, we propose that plakoglobin is a key regulator of cell-cell based viability. That is, when cells are in contact with each other, plakoglobin normally resides at the junctions. When junctions are disrupted, plakoglobin is no longer junctional and cell apoptosis will increase. Determining this has been difficult because regulation of plakoglobin has, up to date, mostly been studied in adherent cells with protein mutation models, which maintains cell-substrate interactions and as a result, may introduce noise into the readouts.

To test our hypothesis, we strategically divided immortalized human keratinocytes into three treatment groups: (1) control, which were cells with both cell-cell and cell substrate contacts; (2) dispase-lifted, which were cells suspended as an intact cell sheet, so that cells maintained cell-cell contact but lost cell-substrate contact, and (3) trypsinized, which were cells that were trypsinized and maintained in suspension dishes as single cells so that the cells had neither cell-cell nor cell-substrate contact. We characterize dispase-lifted cells that maintain cell-cell, but not cell-substrate, contact and show that the cell sheets exhibit contraction, maintain junctional actin, reinforce cell-cell adhesion, and suppress apoptosis in a plakoglobin-dependent manner.

## Results

### Loss of cell-substrate and cell-cell contact results in reorganization of the actin cytoskeleton

Immortalized human keratinocytes were divided into three treatment groups: control (C), which were confluent cells maintained in a cell culture dish, dispase-lifted (D), which were cells that were grown to confluence and then lifted as an intact cell sheet, so that cells maintained cell-cell contact but lost cell-substrate contact, and trypsinized (T), which were cells that were grown to confluence, trypsinized and then maintained in suspension dishes as single cells so that the cells had neither cell-cell nor cell-substrate contact.

**Figure 1:**
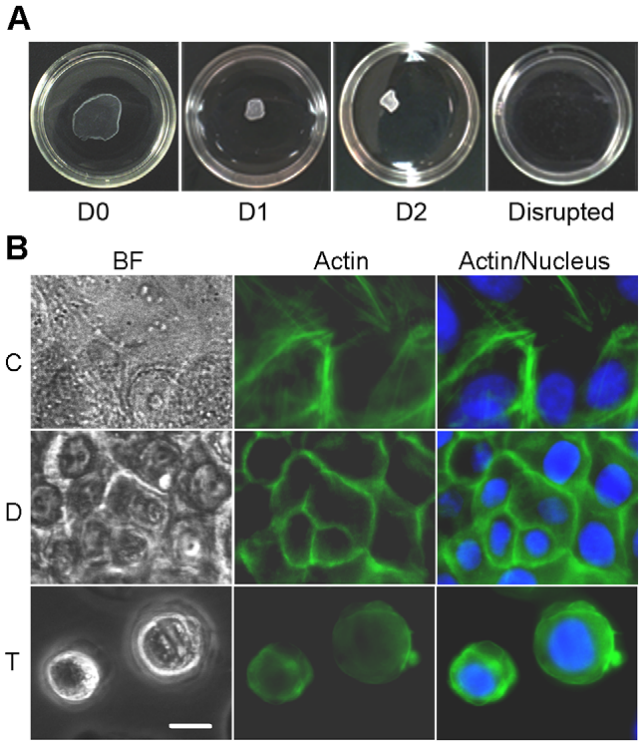
Loss of cell-substrate and cell-cell contact results in reorganization of actin cytoskeleton. (**A**) Images of dispase-lifted cell sheets at zero, one and two days post-dispase lifting, and disrupted by the mechanical shear test, in 35 mm dishes. (**B**) Immortalized human keratinocytes were divided cells into three treatment groups: control (C), confluent cells that maintained in a cell culture dish, had both cell-cell and cell-substrate contact; dispase-lifted (D), confluent cells that lifted as an intact cell sheet, maintained cell-cell contact but lost cell-substrate contact, and trypsinized (T), cells that trypsinized and suspended as single cells, had neither cell-cell nor cell-substrate contact. Representative images of bright field (left panels), actin stain (middle panels), and actin merged with nucleus stain (right panels) in untreated control cells (top panels), dispase-lifted cell sheet (middle panels), and trypsinized cells (bottom panels) show attenuation or loss of non-junctional stress fibers in the dispase-lifted cell sheet and loss of actin organization in the trypsinized cells. Further, cells in the dispase-lifted cell sheet shrunk in the horizontal plane upon release from the substrate. Green: actin, Blue: nucleus. Scale bar: 10 um.

When performing the dispase-lifting assay, the cell sheets showed contraction immediately on separation from the substrate, followed by further contraction one day after separation from the substrate (Figure 1A). A second day of incubation yielded no significant further contraction.

When adhering to the substratum, cells generate stress fibers and adhesion plaques, which allow cells to maintain their spread morphology. To determine (1) the extent of actin reorganization during trypsinization or dispase lifting and (2) whether dispase lifting affects cell spreading, control, dispase-lifted and trypsinized cells were stained for actin immediately after enzyme treatment (i.e., at zero days). Control cells exhibited cortical localization of actin as well as stress fibers through the interior of the cells (Figure 1B). Dispase-lifted cells in the floating sheet exhibited primarily cortical localization of actin, and displayed clearly smaller in-plane cross-sectional areas compared to control cells, suggesting that spreading was reduced on separation from the substrate, consistent with the contraction observed in the whole cell sheet. Trypsinized cells exhibited primarily diffuse staining with no consistent localization of actin within the cell, indicating a nearly complete disruption of actin organization within the cell. Thus, both dispase-lifting and trypsinization alters actin distribution within the cell.

### Cell-cell adhesion strength increases on loss of cell-substrate contact

**Figure 2:**
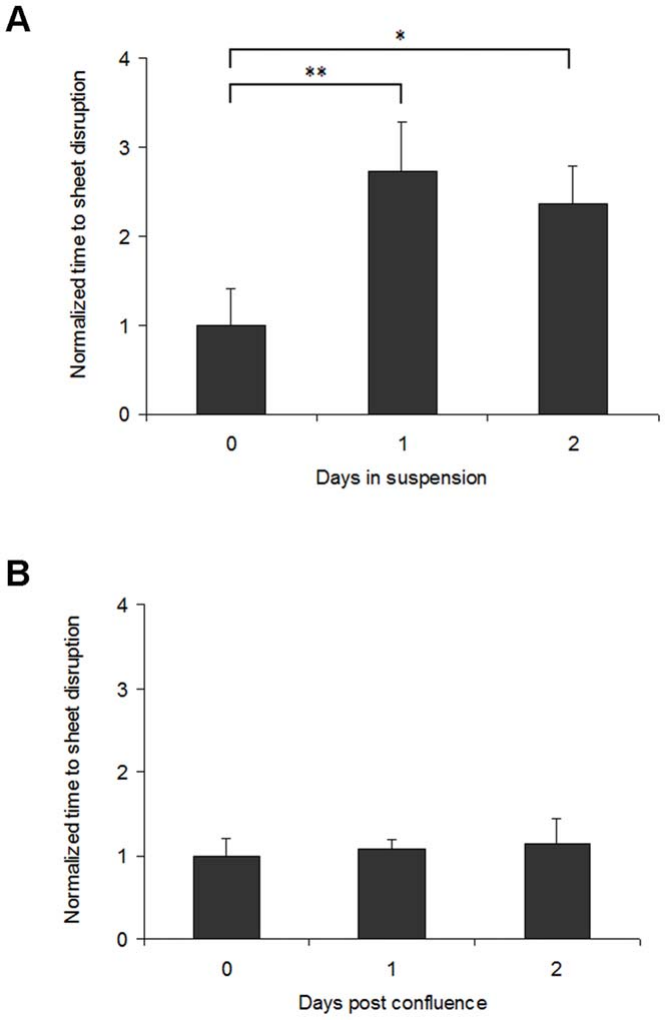
Cell-cell adhesion strength increases on loss of cell-substrate contact. (**A**) Quantification of the strength of cell sheets suspended for zero, one or two days post dispase-lifting. Cell-cell adhesion strength increases significantly from zero to one day, but not from one to two days post dispase-treatment. The y axis represents relative time to sheet disruption, i.e. sheet strength, with values normalized to the time (100%) at zero days. (**B**) The change in strength of the suspended cell sheets is independent of post-confluence effects. *p<0.05 and ** p<0.01 relative to the control at zero days.

Because dispase-lifted cell sheets maintain actin at cell-cell junctions, we next assessed whether cell-cell adhesion is affected by culturing the cell sheets in suspension. To quantify the relative cell-cell adhesion strength of the dispase-lifted cell sheets, a mechanical shearing test, adapted from similar protocols [11], [12], [13], [14], was performed and the amount of time necessary to disrupt the dispase-lifted cell sheet was recorded, with increased disruption time indicating increased cell-cell adhesion strength. Cell-cell adhesion strength increased significantly from day zero to day one post dispase-lifting, but no further from one to two days post dispase-lifting. Specifically, shorter disruption times were measured from day zero sheets (immediately post dispase lifting) compared to sheets lifted and kept in suspension for one and two days post dispase-lifting (p<0.01, day zero versus day one; p<0.05, day zero versus day two, Figure 2A). There was no significant difference in cell-cell adhesion strength between day one and day two (p>0.05), suggesting that after loss of cell-substrate contact, there is fast, but not immediate, reinforcement of cell-cell adhesion strength.

Next, we confirmed that the change in adhesion strength of the dispase-lifted cell sheets is independent of post-confluence effects. Because enhanced cell sheet strength may arise from post-confluence effects, such as overcrowding, cells grown to confluence but maintained while attached to the cell culture dish rather than dispase-lifted, to one day and two days after the typical dispase-treatment target time, were used in the same shearing test protocol. There was no difference in post-confluence cell-cell adhesion if the cells are maintained on a substrate; thus, the reinforcement is due to loss of cell-substrate adhesion and not post-confluence factors (Figure 2B, p>0.05).

Because cell sheets are capable of maintaining cohesion to at least two days after lifting from the substrate, we next examined the apoptotic rate of cells cultured in suspension, with and without cell-cell contact, compared to control cells.

### Cell-cell contact suppresses apoptosis in the absence of substrate adhesion

**Figure 3:**
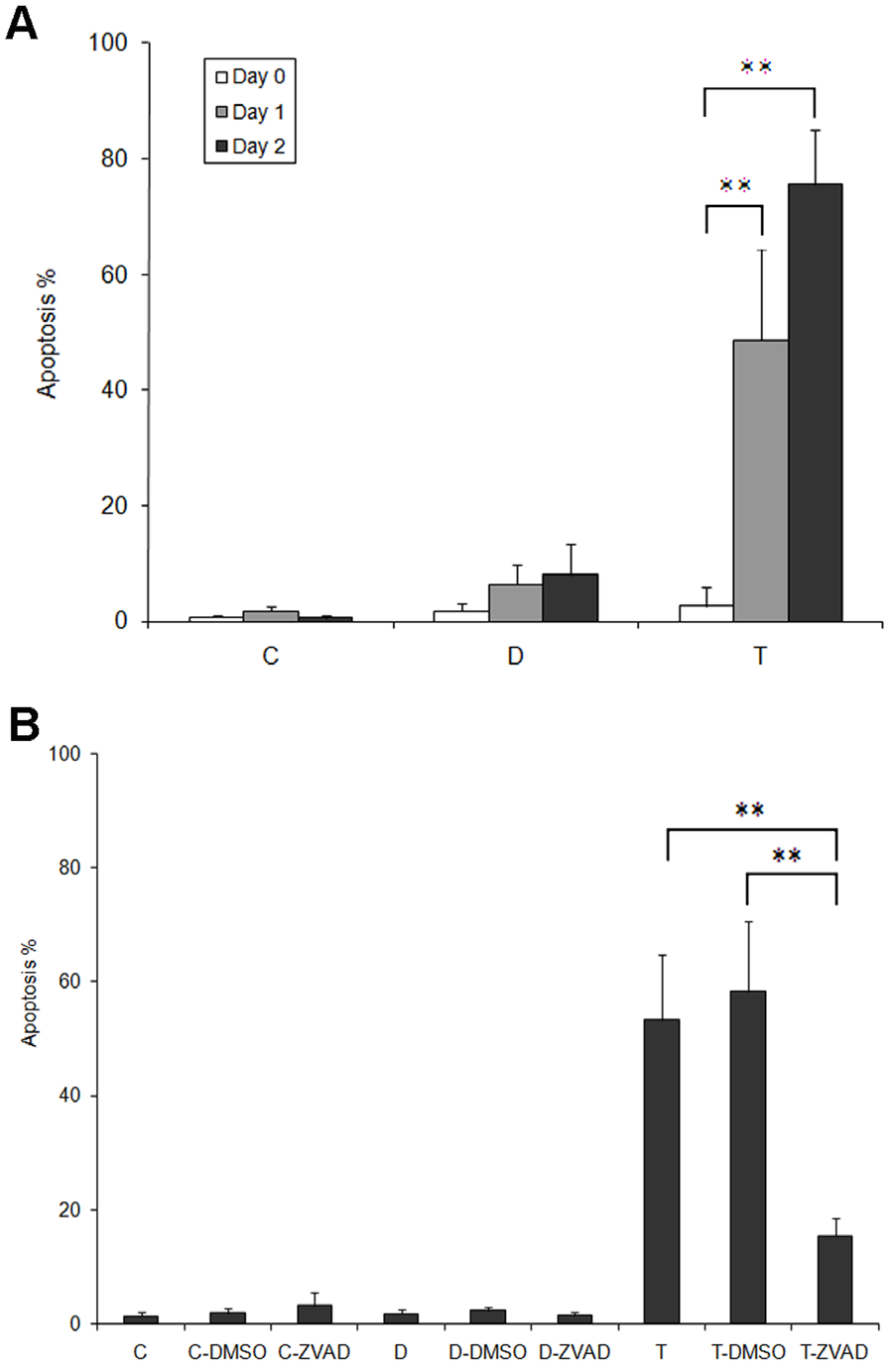
Cell-cell contact suppresses cell death. (**A**) Percentage of cells undergoing apoptosis analyzed at zero, one and two days post treatments for cells left as untreated control (C group), treated with dispase and suspended as cell sheets (D group), or trypsinized and suspended as single cells (T group). Trypsinized cells, lacking both cell-substrate and cell-cell contact, exhibited significantly increased apoptosis after one day post treatment. (**B**) The general caspase inhibitor Z-VAD-FMK (ZVAD) significantly reduced apoptosis in trypsinized single cells. Keratinocytes were grouped into three broad groups: untreated controls (C group, lane 1–3), dispase-lifted and suspended as sheets for one day (D group, lane 4–6), and trypsinized and suspended as single cells for one day (T group, lane 7–9). DMSO was used as a vehicle control. ** p<0.01.

Cells were assayed for apoptosis at three time points: immediately, one and two days post treatment (C0, C1 and C2 for control cells, D0, D1and D2 for cells in dispase-lifted sheet and T0, T1, and T2 for trypsinized, suspended single cells, respectively). Immediately after treatment, the number of apoptotic cells remains statistically unchanged (Figure 3A, apoptosis percentages: 0.65% for control at C0, 1.73% for dispase at D0, and 3.00% for trypsinization at T0, p>0.05). After one day post treatment, a significant percentage of trypsinized cells were apoptotic, whereas controls cells remained overwhelmingly viable (apoptosis percentages: 1.12% for control at C1, 6.40% for dispase at D1, and 48.7% for trypsinization at T1, p<0.01, C1 versus T1). The dispase lifted cells did not exhibit statistically significantly increased apoptosis at one day (p>0.05, C1 versus D1).

After two days post treatment, an even higher percentage of trypsinized cells were positive for apoptosis (Figure 3A, apoptosis percentages: 0.61% for control at C2, 8.17% for dispase at D2, 75.60% for trypsinization at T2, p<0.01, C2 versus T2). Again, the dispase lifted cells did not exhibit increased apoptosis compared to controls (p>0.05, C2 versus D2).

To determine whether the apoptosis is caspase-dependent, cells were treated with a general caspase inhibitor Z-VAD-FMK (ZVAD). DMSO was used as a vehicle control. Keratinocytes were grown to full confluency, and then were grouped into three conditions: controls (C), dispase-lifted (D), and trypsinized (T) of which dispase-lifted and trypsinized cells were suspended for one day, with each group consisting of untreated, DMSO-treated and ZVAD-treated cells. There were no significant differences within the three treatments of the control and dispase groups. Cells trypsinized and suspended for one day exhibited increased apoptosis (Figure 3B, p<0.005 versus control in normal media, p<0.001 versus control in DMSO-supplemented media). Cells dosed with ZVAD exhibited increased apoptosis (p<0.05) but with only a marginal increase in the actual apoptotic rate (from 3.24% to 15.42%). Compared to trypsinization in normal media or in DMSO-supplemented media, ZVAD dosing significantly reduced apoptosis (p<0.01).

The results from trypsinization are consistent with previous work [8] indicating cells require some form of contact maintain viability. However, our data demonstrate that cells do not need to be attached to, or allowed to spread on, a substrate to remain viable, so long as there is some degree of cell-cell contact. Further, these data show that apoptosis resulting from loss of contact is mainly, but not totally, caspase-dependent. Because of previous data implicating plakoglobin as a key signaling molecule in ARVC models, we next examined the role of plakoglobin in suppressing apoptosis in the absence of cell-substrate adhesion.

### Plakoglobin translocates to the cytoplasm and nucleus after loss of cell–cell contact

**Figure 4:**
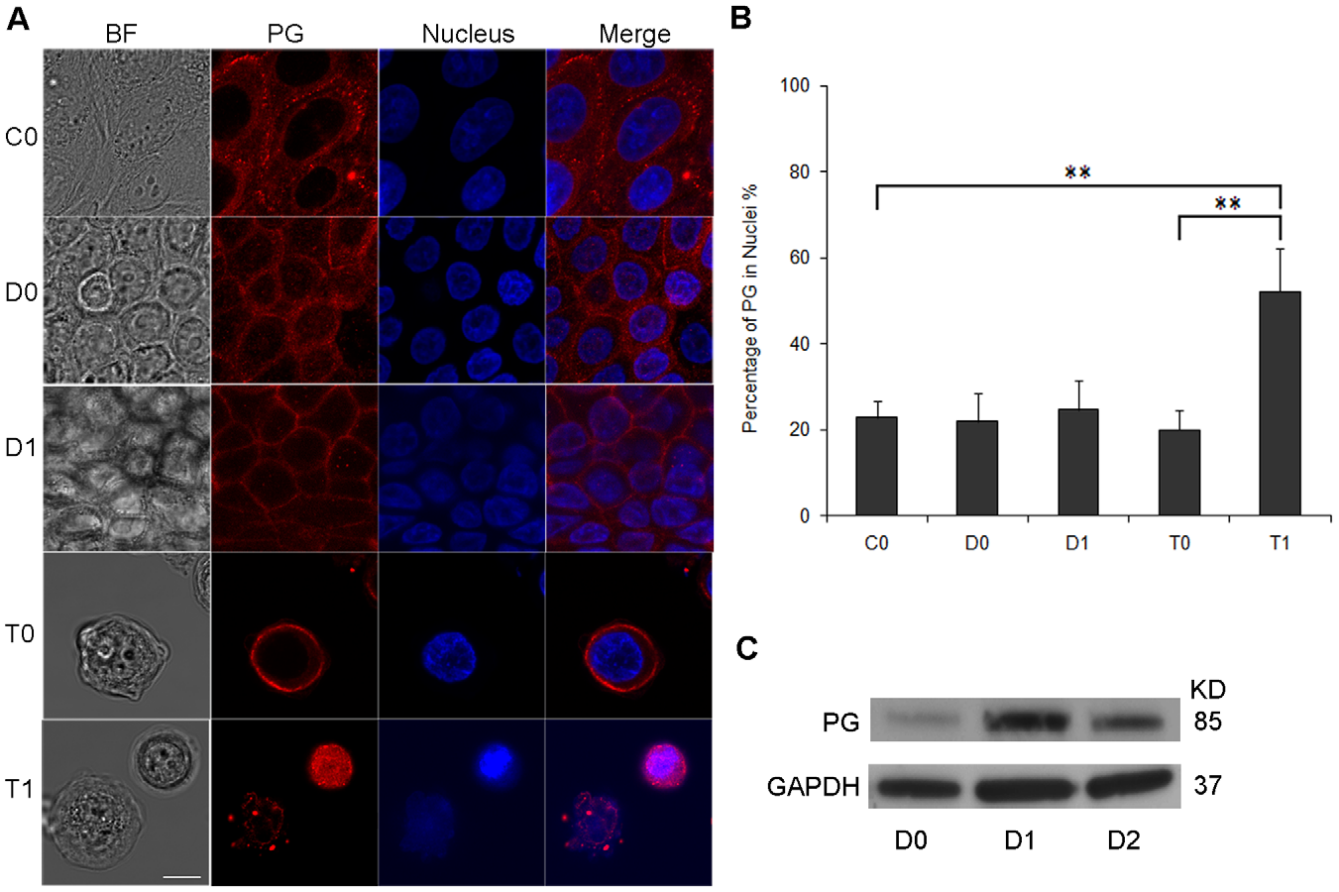
Plakoglobin translocates to the cytoplasm and nucleus after loss of cell–cell contact. (**A**) Plakoglobin localization was assessed using confocal fluorescence microscopy. Keratinocytes were either untreated (C0), treated with dispase and suspended as cell sheet for zero or one days (D0 and D1) or trypsinized and suspended as single cells for zero or one day (T0 and T1). Shown are images of bright field (left panels), plakoglobin stain (second left panels), nuclear stain (second right panels), and plakoglobin merged with nuclear stain (right panels) in untreated control cells (top row), dispase-lifted cell sheet (second and third rows), trypsinized cells (fourth and fifth rows). Plakoglobin was shown predominantly localized to cell junctions in control cells (C0) and the floating cell sheets (D0 and D1). In contrast, plakoglobin exhibited a more diffuse localization into the cytoplasm once cell-cell adhesion was lost (T0), and translocated significantly into the nuclei at one day post trypsinization (T1). Red: plakoglobin (PG), Blue: nucleus. Scale bar: 5 um. (**B**) Quantification of the percentage of plakoglobin in nuclei, showing elevated nuclear plakoglobin in cells trypsinized for one day. ** p<0.01. (**C**) Western blot analysis of dispase-lifted and suspended cell sheets shows plakoglobin level increases significantly from zero to one day post dispase-lifting, but not from one to two days post dispase-lifting. GAPDH was used as a loading control.

Plakoglobin is thought to play a major role in various signaling and structural processes including cardiac and skin cohesion, tumor metastasis and the Wnt-catenin regulatory pathway. To examine the localization of plakoglobin in cells in our three treatment groups (control, dispase-lifted and trypsinized), we performed immunofluorescence staining immediately upon treatment (zero days) and at 24 hours post treatment (one day). Control (untreated) cells exhibited primarily junctional localization of plakoglobin (Figure 4A). Dispase-lifting did not appear to significantly affect plakoglobin distribution. In contrast, trypsinized cells clearly exhibited diminished plakoglobin localization at the cell boundary and increased localization of plakoglobin in the cell cytoplasm, suggesting loss of junctional plakoglobin.

The percentage of plakoglobin overlapping the nuclei were quantified from the confocal immunofluorescence images (Figure 4B, percentages of PG in nuclei: 22.8% for C0, 21.8% for D0, 24.6% for D1, 19.9% for T0, and 52.1% for T1). There was substantially more plakoglobin overlapping the nuclei in cells trypsinized and suspended for one day (p<0.01 for C0 versus T1, p<0.01 for T0 versus T1). CellTracker Green, a cell-permeant, nonspecific fluorescent dye, was used to confirm the nuclear translocation of plakoglobin (Figure S1A). Similar quantification analysis indicated that there were no significant differences for the amount of CellTracker Green in nucleus within the five groups (Figure S1B). Thus, this result demonstrates that plakoglobin, but not CellTracker Green, translocates to nucleus after loss of cell-cell contact.

Western blot analysis shows expression level of plakoglobin in dispase-lifted cell sheets increase significantly from zero to one day post dispase-lifting, but decreases again from one to two days (Figure 4C). Increase in plakoglobin expression above baseline likely correlates to the increase in cell-cell adhesion (Figure 2A), but apparently a limit is reached so that the decrease in plakoglobin from days one to two is not significantly associated with a decrease in cell-cell adhesion.

### Suppression of plakoglobin inhibits apoptosis in trypsinized cells

**Figure 5:**
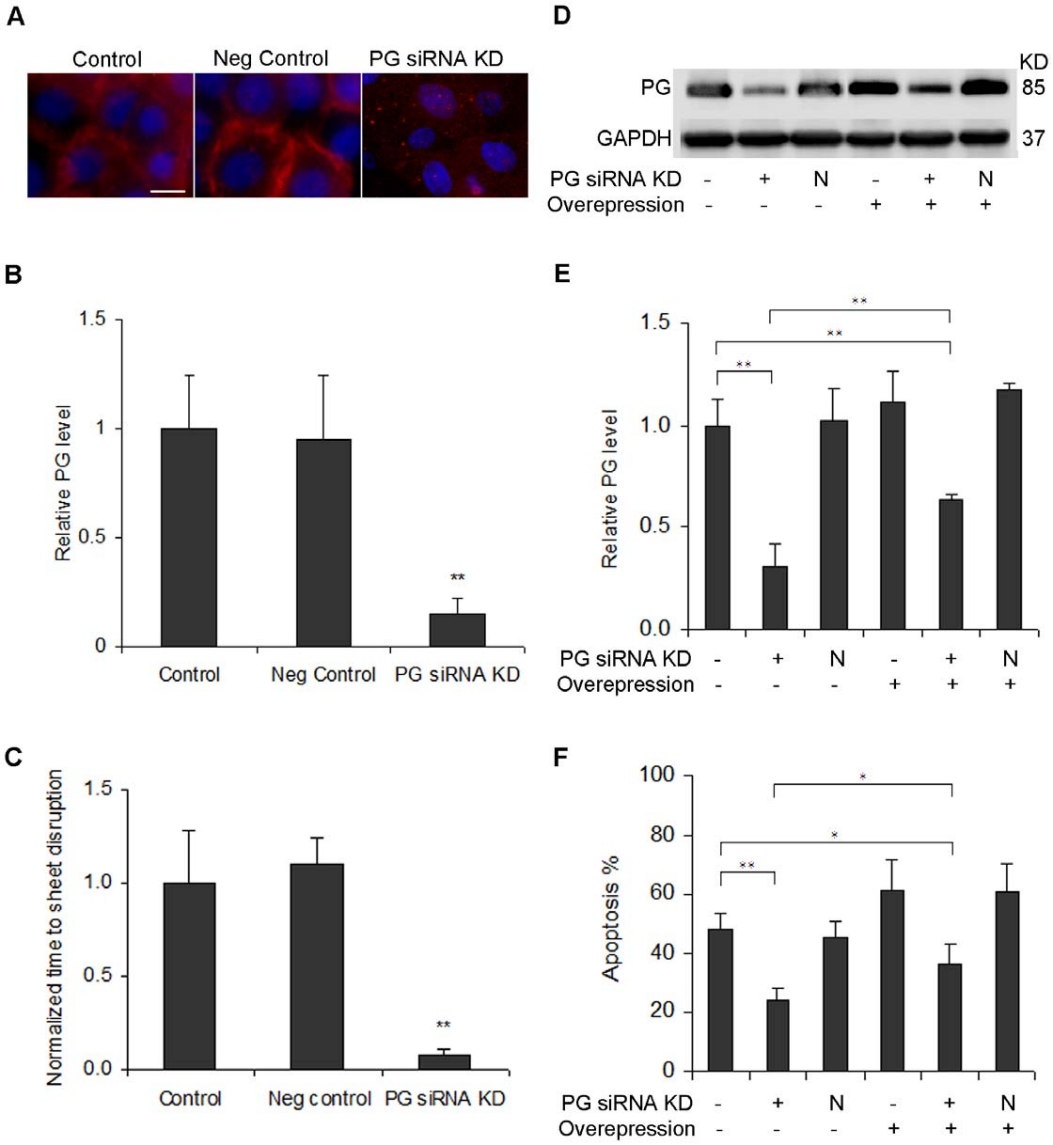
Plakoglobin is a key regulator of the apoptotic pathway. (**A**) Immunofluorescent images of untreated control (left) and plakoglobin knockdown four days after transfection with siRNA (right). Green: plakoglobin, Red: Nucleus. Scale bar: 10 um. (**B**) Quantification of relative plakoglobin levels based on fluorescence images. The y axis represents relative plakoglobin intensity, with values normalized to the untreated control. (**C**) Quantification of the strength of cell sheet transfected with siRNA for plakoglobin (PG siRNA KD) and a negative control (Neg control). Cell sheets were dispase-lifted four days after transfection. The y axis represents relative time to sheet disruption, i.e. sheet strength, with values normalized to the untreated control. (**D**) Western blot analysis of plakoglobin four days after transfection with or without siRNA for plakoglobin (PG siRNA KD), a negative control (N), or plakoglobin overexpression (Overexpression). GAPDH was used as a loading control. (**E**) Quantification of relative plakoglobin level in each western blot lane. The y axis represents relative plakoglobin intensity, with values normalized to the untreated control. (**F**) Apoptosis rate of cells transfected with siRNA for plakoglobin, negative control (N), or plakoglobin overexpression vector. Cells were trypsinized four days after transfection, and then suspended for one day, after which the TUNEL assay was performed. * p<0.05 and ** p<0.01.

To determine whether plakoglobin plays a role in the apoptotic pathway, RNA interference was used to suppress plakoglobin expression. Immunofluorescence images of plakoglobin knockdown four days after siRNA transfection demonstrated diminished plakoglobin expression throughout the cell (Figures 5A and 5B, p<0.005); transfection with the negative control vector showed no significant difference (p>0.05). The strength of the cell sheet transfected with siRNA against plakoglobin decreased significantly compared to the untransfected control (Figure 5C, p<0.01); transfection with the negative control vector showed no significant difference (p>0.05), which correlates well with the changes of plakoglobin level shown in Figure 5A and 5B.

To determine whether plakoglobin is involved in the apoptotic pathway, cells were transiently transfected with siRNA against plakoglobin or a negative control vector. A CMV-driven plakoglobin vector was used to restore plakoglobin expression. Western blot analysis (Figure 5D and 5E) four days post transfection shows a decrease in plakoglobin level with siRNA transfection and a slight increase in plakoglobin level with plakoglobin overexpression. Relative plakoglobin level was normalized to untreated control set as 100%, with 30.7% for plakoglobin siRNA knockdown (p<0.001). The relative plakoglobin level in the cells transfected with the negative control siRNA (102.3%) was similar to the untreated control (p>0.05). Transfection with CMV-driven plakoglobin led to an increase in plakoglobin level (111.0% for CMV-driven plakoglobin alone, 117.3% for CMV-driven plakoglobin vector and negative control vector co-transfection, p>0.05 versus untreated control for both cases). Co-transfection with siRNA for plakoglobin and the CMV-driven plakoglobin vector resulted in a significant increase in plakoglobin level compared to transfection with siRNA alone (63.8% for CMV-driven plakoglobin and plakoglobin siRNA co-transfection, p<0.01 versus untreated plakoglobin siRNA alone). However, co-transfection still exhibited decreased plakoglobin level compared to untreated controls (p<0.01), likely due to the overpowering nature of the siRNA.

Next, we assessed the effects of plakoglobin on the apoptotic rate of cells transfected with siRNA for plakoglobin, the negative control vector, the CMV-driven plakoglobin vector, or combinations thereof. Cells were trypsinized four days post transfection, then suspended for an additional day. As shown in Figure 5F, there was a significant decrease in the percentage of cells undergoing apoptosis in the cells suppressing plakoglobin, suggesting that even partial inhibition of plakoglobin is sufficient to rescue approximately half the cells (48.2% apoptosis for untreated control, 24.3% apoptosis for plakoglobin siRNA knockdown, p<0.0005). The percentage of cells undergoing apoptosis in the cells transfected with the negative control siRNA (45.2%) was similar to the untreated control (p>0.05). Transfection with CMV-driven plakoglobin led to a marginal increase in cell death (61.1% apoptosis for CMV-driven plakoglobin alone, 60.6% apoptosis for CMV-driven plakoglobin vector and negative control vector co-transfection, p>0.05 versus untreated control for both cases). Co-transfection with siRNA for plakoglobin and the CMV-driven plakoglobin vector resulted in a significant increase in apoptosis rate compared to transfection with siRNA alone (36.2% apoptosis for CMV-driven plakoglobin and plakoglobin siRNA co-transfection, p<0.05 versus untreated plakoglobin siRNA alone). However, co-transfection still exhibited decreased apoptotic rate compared to untreated controls (p<0.05), likely due to the overpowering nature of the siRNA. The changes in apoptosis rate are well correlated with the relative expression level of plakoglobin showed in Figure 5D and E. These apoptosis experiments were repeated with a second siRNA against plakoglobin (data not shown), with similar results.

## Discussion

Cell apoptosis is induced by trypsinization, but not dispase-lifting, suggesting that cell-substrate interactions, and spreading of cells, are not the ultimate regulators of cell viability. Further, cohesive cell sheets lacking adhesive substrates maintain or reinforce the junctional localization of actin and plakoglobin. Suppression of endogenous plakoglobin protected cells from trypsinization-induced apoptosis, suggesting that plakoglobin deficiency reduces cell susceptibility to apoptosis.

Cell-matrix interactions have been widely used as a standard model system for studying cellular adhesion. Cell attachment and spreading on the extracellular matrix (ECM) through integrins and focal adhesions were found to govern cell vitality [8], [15], [16], [17]. We show that in the absence of cell-ECM/substrate interactions and active spreading, intercellular junctions become a major control mechanism. One possible reason that cell junctions are so important is that junctions are vital information processing centers where clustered molecules can exchange biochemical and mechanical information among adjacent cells. When those regulatory and signaling molecules connect and communicate along the integrated adhesion site-cytoskeleton networks, individual cells may contribute to tissue-wide decision-making by becoming part of a ‘global’ network, even if there is no ECM.

Most, if not all, adhesion sites likely contribute to cell regulation. Provided that properly organized cytoskeleton networks form and are anchored at adhesion sites, either cell-ECM adhesion or cell-cell adhesion is sufficient to maintain cell viability. Isolated cells resulting from trypsinization and cells that were allowed to attach, but prohibited from spreading via micropatterned islands,[8] induced apoptosis. In both cases, cell spreading is highly restricted or nonexistent, and the cytoskeleton is likely disorganized, which render the cell vulnerable. In the case of the dispase-lifted cell sheet, cell spreading is also highly restricted, and a significant part of the internal cytoskeleton is disrupted, but the death rate is extremely low. Thus, we suggest that it is not cell spreading itself, but organized adhesion site-cytoskeleton organization that controls cell life and death.

There is emerging evidence that plakoglobin may participate in the Wnt/LEF/TCF signaling pathway [18], [19], [20]. Plakoglobin has the capacity to translocate to the nucleus and regulate Wnt signaling by competing with β-catenin, affecting β--catenin localization, accumulation and/or function [21], [22]. Given the relative ubiquity of plakoglobin in a broad variety of tissues, it is likely that our conclusion that cell junctions are key to cell fate regulation can be generalized to all contacting cells in multicellular organisms, with perhaps different junctional proteins participating in those tissues lacking plakoglobin. Indeed, it is possible that other junctional proteins, including other desmosomal proteins, may participate in similar, perhaps overlapping, regulatory pathways. We note that this study focuses on keratinocytes, but since keratinocytes are normally adherent, our results are likely general, and we have preliminary evidence that other cell types exhibit similar behavior. Additionally, other studies are consistent with these results.

Almost all tumor cells tumor cells originate in the epithelium and metastasize as single cells [23]. The vast majority of tumor cells that make their way into bloodstream die off quickly, usually within a few hours of leaving the tumor [23]. Expression levels of plakoglobin is often reduced or absent in invasive cancer cells [24], [25], [26], [27], [28], [29], which likely result in weakened cell-cell adhesion and enhanced migratory capacity [6], [12]. However, we show here that plakoglobin may have a more vital role – cells that detach and enter the bloodstream with reduced plakoglobin may be protected from apoptotic effects, and thus lead to enhanced metastatic capabilities. Our findings could provide an additional mechanism underlying the poorer prognosis associated with tumors expressing diminished plakoglobin [30], [31], [32], [33].

Many adhesion defect diseases, such as pemphigus vulgaris (PV) and arrhythmogenic right ventricular cardiomyopathy (ARVC) are thought to be caused in part by alterations in cell junctions. The mechanisms behind these diseases are a source of active investigation but may rely on the fact that diminished cell-cell adhesion leads to increased susceptibility of cell separation, leading to altered junctional protein localization and possibly the engagement of apoptotic pathways. For example, models of ARVC have been shown to exhibit nuclear plakoglobin localization, increased apoptosis and altered cell-cell adhesion [9], [13]. Thus we show here a unifying mechanism for this class of disease, which may eventually be expanded to include muscular dystrophy and other adhesion defect conditions.

Tissue formation depends critically on the ability of cells to form specific contacts with each other [1]. During development, cell-cell interactions are likely far more important than cell-substrate interactions, given the relatively isolated environment of the embryo. In fact, culturing stem cells to induce specific differentiation sometimes requires using a hanging drop method, both to eliminate a hard adhesive substrate and to provide a more three-dimensional environment for differentiation [34]. Understanding the mechanism underlying cell-cell interactions is vital, but difficult, due to the intricacies working with suspended cell masses. Our study represents an important first step towards explaining how cells communicate in a more realistic environment compared to the commonly used in vitro tools. As such, this approach may be a useful supplement to the growing work in developing three-dimensional scaffolds using novel materials.

This study demonstrates that cell junctions are a fundamental control mechanism in governing cell life and that junctional protein plakoglobin is a key regulator of this process. These findings could have significant implications for understanding cell adhesion, modeling disease progression, developing therapeutics and improving the viability of tissue engineering protocols.

## Materials and Methods

### Cell culture and reagents

Immortalized human keratinocytes were maintained as described elsewhere [35], [36]. Briefly, cells were expanded and propagated in keratinocyte serum-free media (abbreviated ker-sfm, media and supplements from Invitrogen, Carlsbad, CA, unless otherwise stated), supplemented with rEGF (0.2 ng/ml) and BPE (25 µg/ml), CaCl_2_ (Sigma, St.Louis, MO, 0.4mM), and penicillin/streptomycin. To grow cells to high confluency, cells were switched to a medium consisting of a 1∶1 mixture of ker-sfm and a medium DF-K, the latter consisting of a 1∶1 mixture of DMEM and Ham's F-12, supplemented with rEGF (0.2 ng/ml) and BPE (25 µg/ml), L-glutamine (1.5 mM) and penicillin/streptomycin.

### Antibodies and reagents

Unless otherwise noted, reagents were purchased from Invitrogen (Carlsbad, CA). The primary mouse monoclonal antibodies anti-plakoglobin (γ-Catenin) and anti-GAPDH (Novus Biologicals) were used for immunoblotting. Immunofluorescence staining was performed using the anti-plakoglobin antibody as the primary and Alexa Fluor 594 goat anti-mouse IgG as the secondary antibody. Alexa Fluor 488 conjugated phalloidin at a concentration of 0.5 ug/ml was used for the actin stain. CellTracker Green CMFDA dye was used at a concentration of 10 µM. Hoechst was used at a concentration of 0.5 µg/ml for nuclear staining. Trypsin-EDTA (0.05% concentration) and 2.4 units/ml dispase in Hanks Balanced Salt Solution was used for cell treatments.

### Fluorescence microscopy

Cells were fixed in 4% paraformaldehyde (Sigma) and then permeabilized with 0.1% triton-X-100 (Sigma). Cells were then incubated in the primary antibody or the phalloidin, CellTracker Green (CTGreen) and Hoechst label for an hour, followed by PBS washes and then, for samples tagged with a primary antibody, a secondary antibody incubation for another hour, washed again and then imaged.

Microscopy was performed at room temperature using an Olympus IX-81 inverted fluorescence microscope and images were acquired using an Olympus UPlanFL 10x NA 0.13 and Olympus LCPlanFL 40x NA 0.60 objective lens, and an Orca CCD (Hamamatsu, Bridgewater, NJ, model C10600) camera using MetaMorph Software. Additional images were acquired using an Olympus FV10 Confocal microscope, an Olympus OPLFLN 40X O NA 1.3 objective len, and Olympus FV10-ABW Software. Images were processed using ImageJ (version 1.43u for Windows; National Institutes of Health) and scaled down in Photoshop (Adobe) to prepare the final figures. Images were brightness/contrast enhanced and fluorescent colors were added for clarity. Relative nuclear and overall plakoglobin intensities were quantified by ImageJ, with values normalized to the signal (100%) at untreated control. Imaging conditions were taken identical and analyses were done in the raw fluorescence images.

### Assessment of apoptosis using a TUNEL assay

Keratinocytes were grown to full confluency in 35 mm culture dishes, and then were grouped into three conditions: (1) untreated controls, which were cultured in ker-sfm media, (2) trypsinized, which were treated with a Trypsin-EDTA solution, suspended in ker-sfm media and transferred to 35 mm suspension culture dishes, or (3) dispase-lifted, which were treated with dispase, resuspended in ker-sfm medium and transferred to 35 mm suspension culture dish as an intact cell sheet. An *in situ* cell death detection kit (Roche Diagnostics, Basel, Switzerland) that measures TdT-mediated dUTP nick end labeling (TUNEL) was used in the apoptosis assays according to the manufacturer's instructions.

### Dispase-based cell sheet lifting assay

A dispase-based lifting assay was performed to test cell sheet, and thus cell-cell adhesive, strength, adapted from similar protocols [11], [12], [13], [14]. Cells grown to full confluence in 35 mm culture dishes were treated with dispase until the monolayer lifted from the dish as an intact cell sheet. The cell sheets were then suspended in ker-sfm media for zero, one or two days prior to the lifting assay. Cell sheets were carefully transferred to 50 ml tubes containing 6ml of ker-sfm media, then vortexed at a fixed setting at 10 second intervals. The earliest time for which the cell sheet was completely disrupted, as assessed visually, was recorded for each sheet. Please refer to published protocols [11], [12], [13], [14] for more technical details. Digital images were captured at room temperature with a Canon PowerShot SD1100 IS.

### Caspase inhibition assay

To determine whether the apoptosis is caspase-dependent, cells were treated with a general caspase inhibitor Z-VAD-FMK at a final concentration of 20 um (BD Pharmingen, San Diego, CA). Keratinocytes were grown to full confluency in 35 mm culture dishes, and then were grouped into three conditions: controls, which were cultured in ker-sfm media, dispase-lifted, which were cells suspended as an intact cell sheet, and trypsinized, which were treated with a Trypsin-EDTA solution, suspended in ker-sfm media and transferred to 35 mm suspension culture dishes. DMSO was used as a vehicle control. The TUNEL assay was used to determine the apoptosis rate according to the manufacturer's instructions.

### siRNA knockdown and plasmid overexpression of plakoglobin

Two Silencer® Pre-designed siRNA for plakoglobin (first siRNA sequence 5′-3′: GGGCAUCAUGGAGGAGGAUtt; second siRNA sequence 5′-3′: CCAUCGGCUUGAUCAGGAAtt), a negative control siRNA (Invitrogen) and an overexpression vector pCMV6-XL5 (OriGene, Rockville MD) were used to assess the effects of inhibition and overexpression of plakoglobin on cell survival. Cells were transfected with siRNA using Lipofectamine 2000 (Invitrogen), according to the manufacturer's suggestions. Transfection was performed using 100 pmol siRNA oligomer or 100 pmol DNA plasmid and 6 ul Lipofectamine 2000 for each sample in a 35 mm dish. Other size formats were scaled up or down accordingly. Cells were lysed and analyzed for plakoglobin by immunoblotting four days after transfection. Alternatively, cells were trypsinized 4 days after transfection, then suspended in ker-sfm media in 35 mm suspension culture dishes for one additional day, after which the apoptosis rate of transfected cells were analyzed using the TUNEL assay as described.

### Immunoblotting

Total protein concentration was determined by Bradford assay (Sigma). Soluble fractions containing equal amounts of total protein were separated using SDS polyacrylamide gel electrophoresis and transferred onto PVDF membranes (Millipore, Billerica, MA). Immunoblotting was performed using mouse anti-human antibodies diluted in TBS with 1% w/v BSA and 0.1% v/v Tween-20 (Sigma) at the following dilutions: anti-Plakoglobin 1∶2000, anti-GAPDH 1∶1,000, and horseradish peroxidase conjugated goat anti-mouse secondary antibody 1∶1,000. Blots were developed with ECL reagents (Perkin Elmer, Waltham, MA) and imaged using a FUJI imaging unit (Fujifilm, Stamford, CT). Relative plakoglobin intensity in immunoblotting images was quantified by ImageJ, with values normalized to GAPDH of each matching blot lane and the signal (100%) at untreated control.

### Statistical analysis

Data are expressed as the mean ± SD. Differences among more than 2 groups were compared by ANOVA and Tukey's post-test as appropriate. A value of p<0.05 was considered significant, with each group having sample sizes n ≥3.

## Supporting Information

Figure S1 **CellTracker Green doesn**'**t translocate to the nucleus after loss of cell–cell contact.** (**A**) CellTracker Green (CTGreen) localization was assessed using confocal fluorescence microscopy. Keratinocytes were either untreated (C0), treated with dispase and suspended as cell sheet for zero or one days (D0 and D1) or trypsinized and suspended as single cells for zero or one day (T0 and T1). Shown are images of bright field (left panels), CTGreen stain (second left panels), nuclear stain (second right panels) and CTGreen merged with nuclear stain (right panels) in control cells (top row), dispase-lifted cell sheet (second and third rows), trypsinized cells (fourth and fifth rows). Green: CTGreen, Blue: nucleus. Scale bar: 5 µm. (**B**) Quantification of the percentage of CTGreen in nuclei, showing no significant difference of nuclear CTGreen among the groups.
(TIF)
