# Supplementary figures and images for: Cell-Cell Contact Preserves Cell Viability via Plakoglobin
Source: PLoS One. 2011 Oct 28;6(10):e27064. doi: 10.1371/journal.pone.0027064 (PMC3203941; doi:10.1371/journal.pone.0027064)

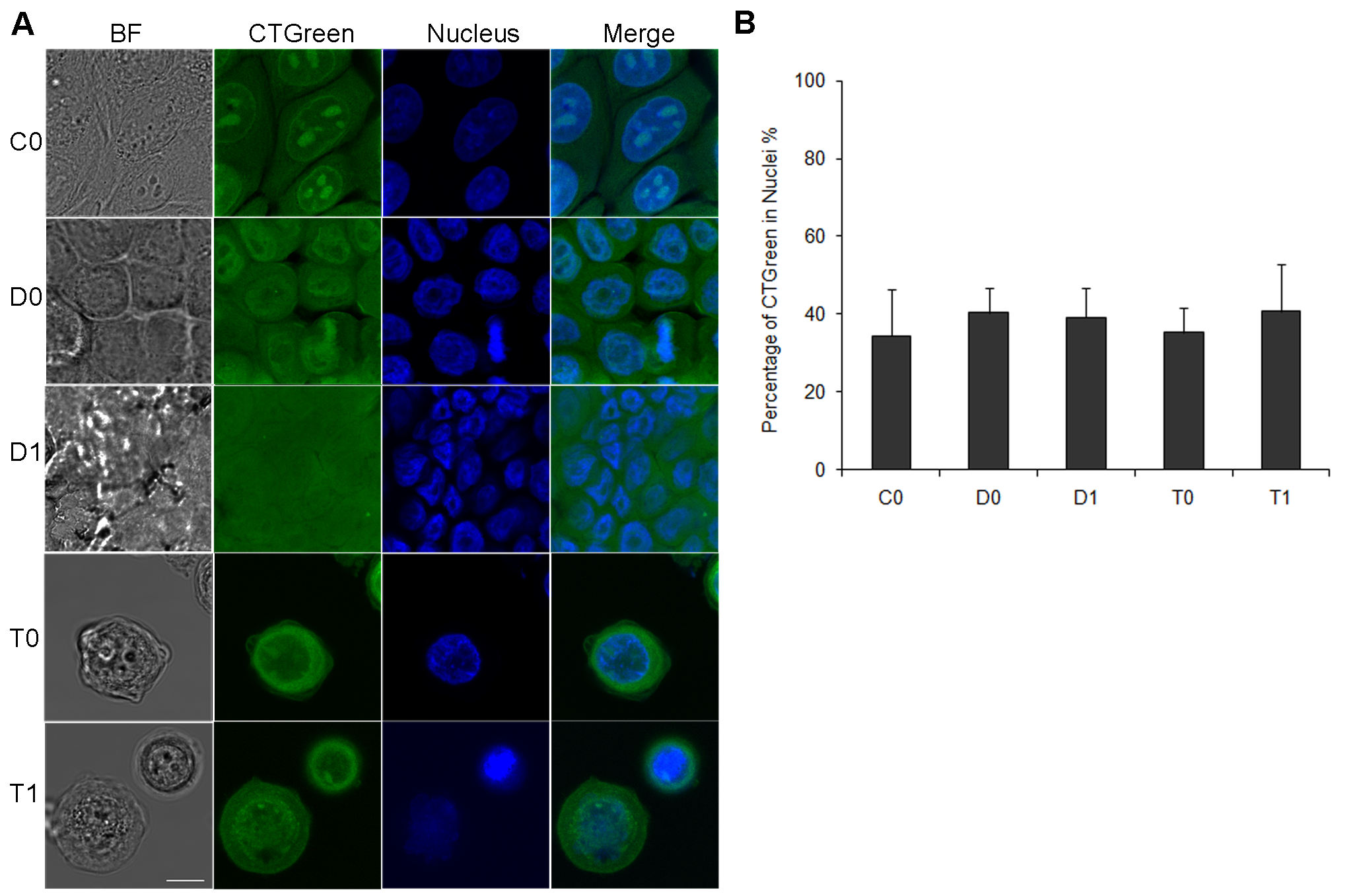

Supplement: Figure S1 — CellTracker Green doesn't translocate to the nucleus after loss of cell–cell contact. (A) CellTracker Green (CTGreen) localization was assessed using confocal fluorescence microscopy. Keratinocytes were either untreated (C0), treated with dispase and suspended as cell sheet for zero or one days (D0 and D1) or trypsinized and suspended as single cells for zero or one day (T0 and T1). Shown are images of bright field (left panels), CTGreen stain (second left panels), nuclear stain (second right panels) and CTGreen merged with nuclear stain (right panels) in control cells (top row), dispase-lifted cell sheet (second and third rows), trypsinized cells (fourth and fifth rows). Green: CTGreen, Blue: nucleus. Scale bar: 5 µm. (B) Quantification of the percentage of CTGreen in nuclei, showing no significant difference of nuclear CTGreen among the groups. (TIF) [file pone.0027064.s001.tif]
